# Chemical Modification Mechanism of SiC Substrates in Electrical Discharge Machining

**DOI:** 10.3390/mi17050618

**Published:** 2026-05-18

**Authors:** Qiufa Luo, Gu Li, Ningchang Wang, Sirong Wang, Jing Lu, Congming Ke

**Affiliations:** 1 Institute of Manufacturing Engineering, Huaqiao University, Xiamen 361021, China; 18672220323@163.com (G.L.); 17513264114@163.com (S.W.); cmke@hqu.edu.cn (C.K.); 2 National & Local Joint Engineering Research Center for Intelligent Manufacturing Technology of Brittle Materials Products, Huaqiao University, Xiamen 361021, China; 3 State Key Laboratory of High Performance Tools, Xiamen 361021, China; 4 State Key Laboratory of High Performance Tools, Zhengzhou Abrasives Grinding Research Institute Co., Ltd., Zhengzhou 450001, China; wangningchang@126.com

**Keywords:** SiC substrate, electro-discharge chemical modification, interface temperature field, material removal behavior, modified layer

## Abstract

Electrical discharge machining (EDM) is an efficient method for processing silicon carbide (SiC) substrates. However, the chemical modification mechanism of SiC substrates in the EDM process remains not fully elucidated. To clarify the material removal mechanism of SiC substrates in EDM, this study investigated the behaviors of SiC substrates under different discharge conditions through experimental analysis and interface temperature field simulation. Results indicate that the SiC substrates sequentially exhibit characteristic morphologies of surface oxidation, thermal decomposition, and fracture as discharge energy increases. A discolored layer composed of amorphous SiO_2_ is formed on the SiC surface in low-discharge energy. Crystalline silicon and graphitic carbon are generated from the thermal decomposition of SiC substrates in high-discharge energy. Excessively high discharge energy induces the breakdown of SiC substrates. A critical temperature threshold is identified that delineates the initiation of prominent thermal oxidation on the SiC surface. Temperature field simulations further reveal the correlation between EDM parameters and interfacial temperature variations, along with the mechanisms of material removal driven by thermal diffusion. This study deepens the fundamental understanding of the EDM removal mechanism of SiC substrates and is expected to provide a scientific basis for the efficient material removal of SiC substrates.

## 1. Introduction

Characterized by high thermal stability, exceptional hardness, and superior corrosion resistance [1,2,3], SiC underpins its importance in energy, electronics, and rail transit fields [4,5]. However, formidable challenges to machining SiC efficiently and precisely are presented with the growing demand for larger substrates.

Chemical mechanical polishing (CMP) is currently the most widely utilized final planarization technology [6,7,8]. However, due to the high hardness and chemical inertness of SiC, as well as the trend toward larger substrate sizes, achieving an ultra-precise surface with a roughness of less than 0.3 nm often requires a polishing time of more than six hours, which leads to low processing efficiency and high production costs [9,10]. To address this challenge, external energy field-assisted machining technologies have become a research focus. Electrical discharge machining (EDM) demonstrates unique advantages by achieving efficient removal of conductive-type hard materials through a non-contact thermoelectric effect [11,12,13,14]. The high temperatures generated during the EDM process can instantaneously change the physical state of materials, inducing chemical reactions such as thermal decomposition and oxidation, thereby significantly increasing the material removal rate (MRR) and shortening overall processing time [15].

In traditional processes, EDM has been applied to the drilling [16,17,18,19], slicing [20,21], and milling [22] of SiC materials. However, craters, recrystallized materials, cracks and micropores are generated on the machined surface under the action of thermal energy produced during the EDM process [16]. Key parameters in the EDM process have been focused on to achieve high-quality machining of SiC materials, such as electrode characteristics [23,24,25,26,27], electrical parameters [28,29,30], and dielectric fluid characteristics [31,32]. Mohan et al. [23] conducted investigations on the EDM of Al-SiC composites and found that brass electrodes and positive polarity exhibited superior performance in terms of MRR, whereas copper electrodes and negative polarity were more advantageous in reducing tool wear rate and surface roughness. Yoo et al. [28] studied the effect of single discharge energy on yttrium nitride-reinforced silicon carbide (YN-SiC), pointing out that the optimal machining accuracy of YN-SiC was achieved when the single discharge energy was controlled at approximately 7 μJ, while a higher MRR was obtained at a discharge energy exceeding 10 μJ. Zhang et al. [31] suggested that the medium type significantly affects the material removal characteristics of EDM, with liquid media maintaining a higher pressure at the discharge point and thus achieving a much higher material removal efficiency than gaseous media. In addition, chemical reactions occurring in EDM also affect the machining performance of certain materials. Gong et al. [33] found that a ZrO_2_-SiO_2_ oxide film forms on the surface of Cf-ZrB_2_-SiC during EDM, which can effectively inhibit the shedding of carbon fibers. These studies confirm that EDM exhibits great potential in achieving high-efficiency or high-quality machining of hard and brittle materials simultaneously through reasonable regulation of process parameters.

The application of EDM technology in the manufacture of SiC substrates has emerged as a new research hotspot. Guan et al. [34] developed a novel non-contact machining method for semiconductor substrates and achieved a maximum MRR of 11.2 μm/min for SiC substrates via ultra-short pulse EDM. On this basis, a bipolar EDM method was proposed to realize the synchronous thinning of double SiC substrates [35]. Yan et al. [36] employed Co-bonded polycrystalline diamond (PCD) rods as hybrid tools and realized high-efficiency rough shaping of SiC by electrical discharge machining (EDM). Subsequently, diamond grains protrude from the tool surface due to the erosion of the Co binder phase in PCD during EDM, which realizes the precise removal of the EDM-induced damaged layer. However, most studies have mainly focused on optimizing the EDM parameters to achieve specific objectives, such as improving MRR and reducing surface roughness. Research on the thermochemical reactions of SiC substrates during EDM remains relatively limited. Exploring the chemical reactions involved in the EDM process is expected to further promote the application of EDM in SiC substrate manufacturing.

In this paper, the chemical reaction mechanism of SiC substrates under different discharge conditions was investigated by experimental characterization and multiphysics simulations. A systematic investigation was carried out to establish correlations among EDM parameters, transient interfacial temperature, and resultant surface modifications. Experimental results were verified by COMSOL Multiphysics 6.2 simulations of the temperature field and melt pool morphology, enabling a holistic understanding of the thermochemical processes governing SiC material removal. Such insights offer theoretical guidance for developing high-efficiency, low-damage machining technologies for SiC substrates in advanced semiconductor manufacturing.

## 2. Materials and Methods

### 2.1. Experimental Details

**Figure 1:**
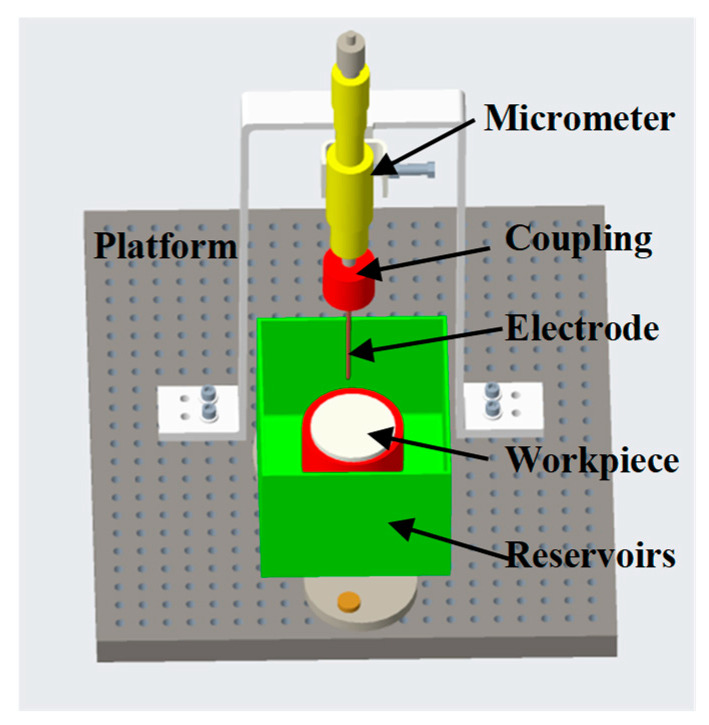
The schematic diagram of the EDM experimental setup.

The schematic diagram of the EDM experimental setup is depicted in Figure 1. The apparatus comprises a working stage, a dielectric fluid reservoir, a workpiece fixture, and a tool electrode assembly. A transistor-based pulse generator (SOYI-22030M, Shanghai Soyi Electronics Co., Ltd., Shanghai City, China) was used as the power supply in the experiments. Specific experimental parameters are presented in Table 1. A standard tapered copper tool electrode (P2.5-L70) was utilized; the effective discharge diameter of the electrode is 0.15 mm. The workpiece was fully submerged in the dielectric medium during EDM. Deionized water was used as the dielectric medium in the experiments unless otherwise specified. The inter-electrode gap was precisely controlled by a digital micrometer with a resolution of 1 µm. The digital micrometer was set to zero when the electrode was in contact with the workpiece. Experiments were conducted on the Si-face (0001) of 4H-SiC substrates. The 4H-SiC substrate is n-type nitrogen doping, with a room-temperature resistivity of 0.015–0.025 Ω·cm and a doping concentration of approximately 2 × 10^19^–4 × 10^19^ cm^−3^.

The surface morphologies and chemical composition of SiC substrates were analyzed using a scanning probe micro-Raman spectrometer (Alpha 300RA, Oxford Instruments, Ulm, Baden-Württemberg, Germany) and an X-ray photoelectron spectrometer (K-alpha, Thermo Fisher, Waltham, MA, USA). Surface roughness measurements were performed using a white light interferometer (NewView 7300, ZYGO, Middlefield, CT, USA). A high-speed infrared thermal imager (ImageIR 5325, InfraTec, Dresden, Saxony, Germany) was used to observe the surface temperature of SiC during the discharge process. A precision power analyzer (WT1806E, YOKOGAWA, Hachioji, Tokyo, Japan) was used to measure the discharge power during the experiment. An electric blast drying oven (101-3S, Changge City Mingtu Machinery Equipment Co., Ltd., Changge City, Henan Province, China) was employed for the ablation experiments.

### 2.2. Simulation Model of Temperature Field

**Figure 2:**
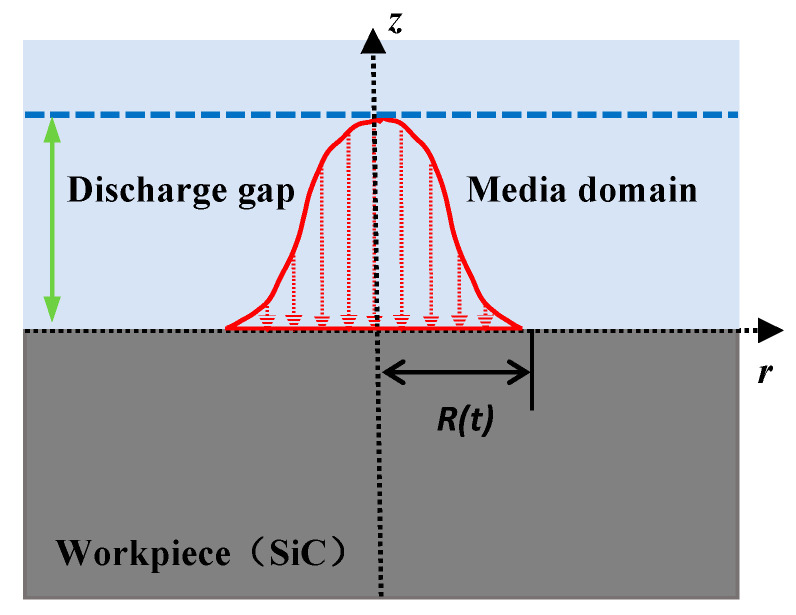
Simulation model of temperature field.

To investigate the thermo-physical processes governing the surface modification of SiC substrates during EDM, a finite element model was established using COMSOL multiphysics. The model simulates the transient temperature evolution and subsequent morphological changes on the workpiece surface resulting from the heat flux of a single plasma channel. As shown in Figure 2, the 2D computational domain comprises a workpiece domain (200 × 60 µm) and an overlying dielectric fluid domain (200 × 20 µm). The domain was discretized using a free triangular mesh, which was significantly refined in the region of heat impact. The mesh consisted of 2616 elements, with a minimum element size of 25 nm and a maximum of 7.4 µm. The present model is intended only for qualitative analysis of temperature field distribution and thermal influence range, rather than accurate quantitative prediction of absolute peak temperature or phase-change behavior.

The heat flux distribution is described by Equation (1) [37].

(1) $$q(r)=\frac{Q}{\pi \times R^{2}}\times e^{\frac{-3r^{2}}{R^{2}}}$$

where *q(r)* is the radial heat flux density (W/m^2^), *Q* is the discharge energy (*J*), *R* is the characteristic radius of the heat source (*m*), and *r* is the radial distance from the discharge center (*m*).

A thermal-flow coupled numerical simulation method was used to investigate the material removal process in EDM. In addition to assuming the fluid as a continuum, the following assumptions were made regarding the effects of the heat source and material properties: (1) Heat radiation were neglected, heat conduction and heat convection were the main modes of heat transfer; (2) Material properties were uniform and isotropic; (3) Material thermal performance and fluid characteristics were functions of temperature; (4) The molten and vaporized material were considered a laminar viscous Newtonian fluid and were incompressible.

To corroborate the experimental results, the simulation parameters selected include the processing time, processing gaps, heat source power, and working medium, which correspond to the experimental parameters of processing times, discharge voltages, discharge gap, and discharge dielectrics, respectively. The conditions adopted for the calculation of the discharge crater solution are presented in Table 2. SiC substrate is used as the anode material in the simulation. The thermophysical properties of air, water and kerosene media are set as shown in Table 3.

## 3. Results

### 3.1. Surface Morphologies of SiC Processed with Different Discharge Parameters

**Figure 3:**
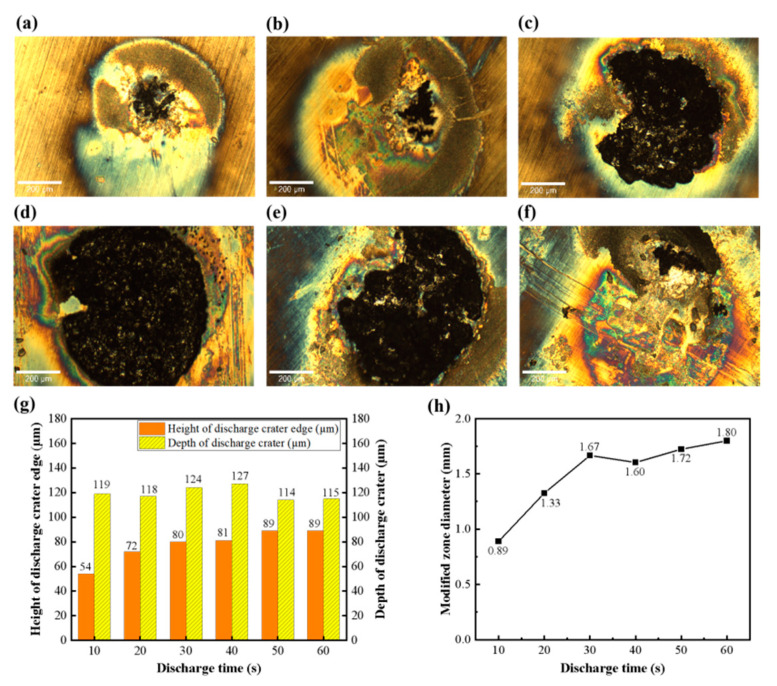
Surface morphologies and characteristics of SiC substrates after EDM under different processing times: (**a**) 10 s, (**b**) 20 s, (**c**) 30 s, (**d**) 40 s, (**e**) 50 s, (**f**) 60 s; (**g**) edge height and depth of the discharge crater; (**h**) modified zone diameter.

Figure 3 illustrates the evolution of the SiC surface morphologies and topographies as a function of processing time. Figure 3a–f shows the surface morphologies of SiC substrates under different processing times. The modified region of SiC was dominated by a discolored area with a small amount of black sediment deposits at a processing time of 10 s. The area of the deposited layer reached a maximum at 40 s and subsequently decreased with further processing. The vertical topographies of SiC substrates under different processing times are depicted in Figure 3g. The height of the discharge crater edge increased until saturation with increasing processing time, while the depth of the discharge crater remained consistently in the range of 114–127 µm throughout the process. Figure 3h presents the maximum diameter of the modified zone under different processing times. The maximum diameter of the modified zone was 0.89 mm at 10 s and then increased rapidly to 1.67 mm at 30 s. Subsequently, the growth rate slowed significantly, and the diameter stabilized at approximately 1.75 mm for processing times from 30 s to 60 s. Collectively, these results indicate that the surface modification process approached saturation at approximately 40 s. Prolonging the processing time had a negligible effect on the area of the modified zone.

**Figure 4:**
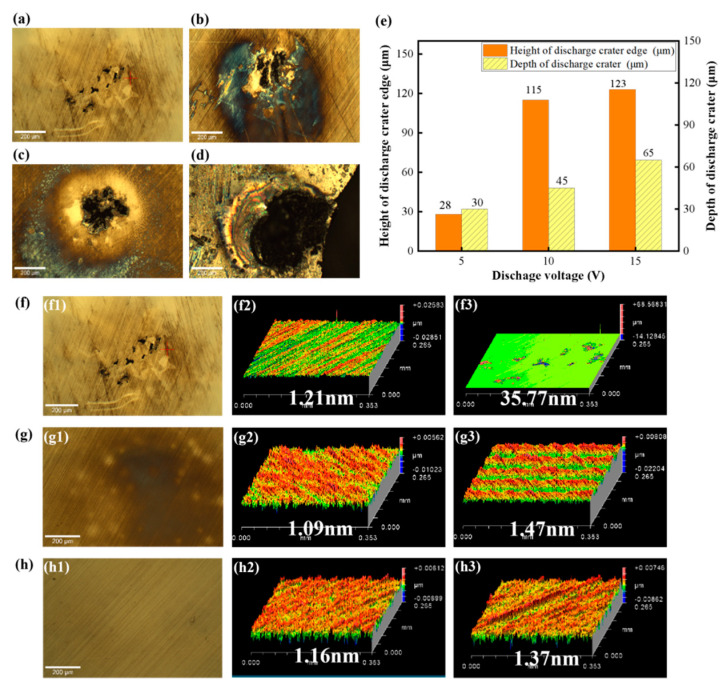
Surface morphologies and characteristics of SiC substrates after EDM at different discharge voltages: (**a**) 5 V, (**b**) 10 V, (**c**) 15 V, (**d**) 20 V; (**e**) edge height and depth of the discharge crater; (**f**–**h**) 5 V, 3 V, 1 V; (**f1**–**h1**) surface morphology, (**f2**–**h2**) surface roughness before EDM, (**f3**–**h3**) surface roughness after EDM.

Figure 4a–d shows the surface morphologies of SiC substrates after EDM at different discharge voltages. The extent of surface modification was slight at a low voltage of 5 V. The affected area on the SiC surface expanded progressively with increasing discharge voltage, and fracture ultimately occurred on the SiC surface at 20 V. As illustrated in Figure 4e, elevating the discharge voltage from 5 V to 20 V resulted in a significant increase in crater depth and edge protrusion height. To identify the critical voltage window for the formation of surface deposits, the modification behavior of SiC substates was investigated at lower discharge voltages of 1 V and 3 V. The surface morphologies and roughness of SiC substrates before and after EDM at 5 V, 3 V and 1 V are presented in Figure 4f–h, respectively. The SiC surface is mainly subject to discoloration with a slight increase in surface roughness at 3 V, which confirms that the threshold voltage for the formation of black agglomerated deposits is 5 V.

**Figure 5:**
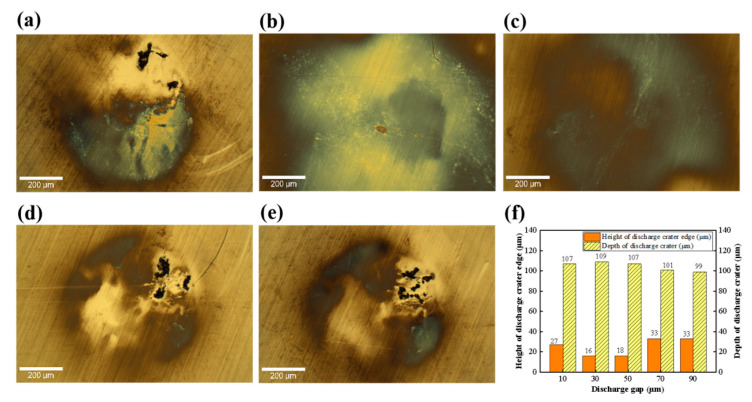
Surface morphologies and characteristics of SiC substrates after EDM under different discharge gaps: surface morphologies at 10 μm (**a**), 30 μm (**b**), 50 μm (**c**), 70 μm (**d**), and 90 μm (**e**); (**f**) edge height and depth of the discharge crater.

Figure 5 shows the surface morphologies and topological structures of SiC substrates after machining with different discharge gaps. Clean and well-defined discharge craters were formed on the machined surface at moderate gaps (30 and 50 μm). However, a prominent redeposited layer was observed on the surface when the gap was too small (10 μm) or excessively large (70 and 90 μm). As illustrated in Figure 5f, the edge protrusion height of the discharge crater first decreased and then increased with increasing discharge gap, while the depth of the discharge crater remained stable at approximately 99–109 μm for all gaps. These indicate that the discharge energy was similar under different discharge gap conditions.

**Figure 6:**
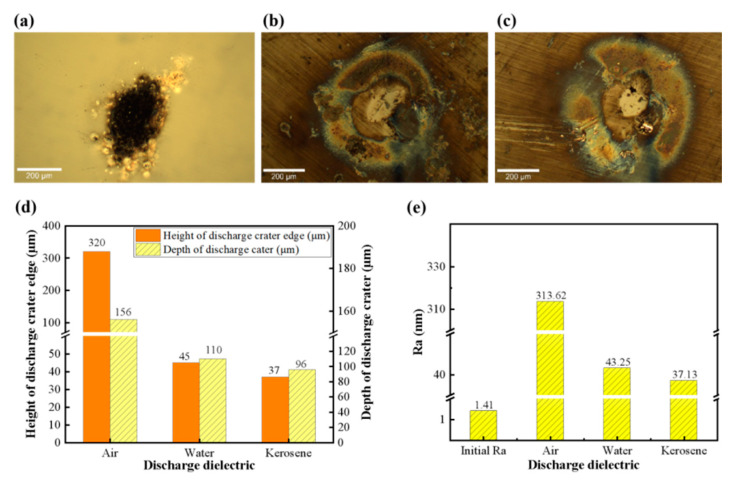
Surface morphologies and characteristics of SiC substrates after EDM in different discharge dielectrics: (**a**) in air, (**b**) in deionized water, (**c**) in kerosene; (**d**) edge height and depth of the discharge crater; (**e**) surface roughness.

Figure 6 presents the surface morphologies and topological structures of SiC substrates after machining in different dielectric media. The morphologies of SiC substrates after EDM in air, deionized water and kerosene are presented in Figure 6a–c. The SiC surface was covered with black sediment deposits after EDM in air, while the SiC surfaces mainly exhibited color changes after EDM in deionized water and kerosene. As shown in Figure 6d, the edge protrusion height and depth of discharge crater on the machined surface in air are significantly larger in size compared with those formed in liquid dielectric media. Among the two liquid media, the degree of SiC surface modification in water is slightly higher than that in the kerosene medium. The surface roughness of the SiC substrate machined in different dielectric media is illustrated in Figure 6e, where the SiC machined in an air environment exhibits the highest surface roughness (313.62 nm). In contrast, the SiC surfaces machined in liquid dielectric media are relatively smooth, with the surface roughness of 43.25 nm and 37.13 nm obtained in water and kerosene media, respectively. These indicate that the dielectric medium exerts a significant influence on the control of discharge energy and deposition on the workpiece surface in EDM.

### 3.2. Composition Analysis of Discharge Area on SiC Surface

**Figure 7:**
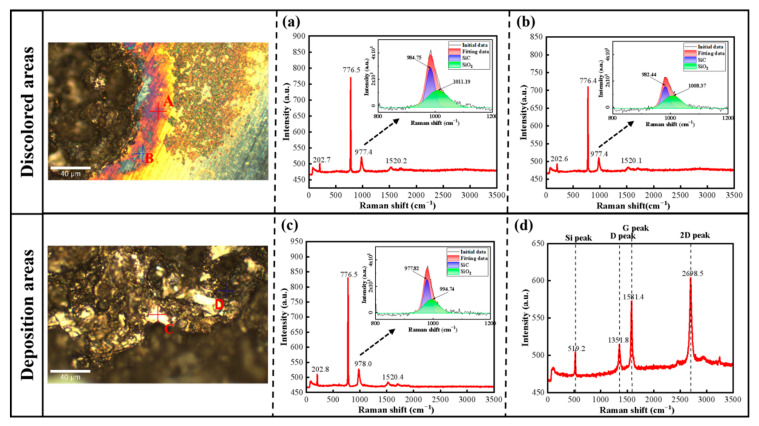
Raman detection results of modified regions on SiC surface: (**a**,**b**) Discolored regions A and B; (**c**,**d**) Deposition regions C and D.

Figure 7 shows the Raman detection results of SiC substrates after EDM. The modified surface consists of two typical characteristic regions: a discolored region and a deposited region. Point Raman analysis was conducted at two positions (A, B) in the discolored region and at another two sites (C, D) in the deposited region, respectively. Figure 7a presents the Raman detection results of Point A in the discolored region. In addition to the characteristic peak of 4H-SiC at 776 cm^−1^, a composite peak attributed to SiC and SiO_2_ was observed at 977.4 cm^−1^. The positions of the new characteristic peaks of SiC and SiO_2_ were at 984.75 cm^−1^ and 1011.19 cm^−1^, respectively, after fitting and peak separation of the original curve. Due to the formation of amorphous SiO_2_ and the oxidized interphase, which induces obvious spectral convolution, the apparent deviation from the standard 964 cm^−1^ LO position. Figure 7b displays the Raman detection results of Point B on the SiC surface, where a composite Raman peak assigned to SiC and SiO_2_ was detected at 977.4 cm^−1^. The positions of the new characteristic peaks of SiC and SiO_2_ were at 982.44 cm^−1^ and 1008.37 cm^−1^, respectively, after fitting and peak separation of the original curve. For the deposited region, Figure 7c shows the Raman detection results of Point C on the SiC wafer surface, with a composite Raman peak attributed to SiC and SiO_2_ observed at 978.0 cm^−1^. The new characteristic peaks of SiC and SiO_2_ were found at 977.82 cm^−1^ and 994.74 cm^−1^, respectively, after fitting and peak separation of the original curve. Notably, the Raman detection results of Point D exhibited distinct characteristic peaks at 519.2 cm^−1^, 1351.8 cm^−1^, 1581.4 cm^−1^ and 2698.5 cm^−1^, where the 519.2 cm^−1^ peak is assigned to crystalline silicon [39,40], and 1351.8 cm^−1^ (D band), 1581.4 cm^−1^ (G band) and 2698.5 cm^−1^ (2D band) peaks correspond to graphitic carbon, as illustrated in Figure 7d. These results confirm that the discolored region is primarily composed of the original SiC substrate with a thin surface SiO_2_ layer, while the deposited region consists of crystalline silicon and graphitic carbon decomposed from the SiC substrate.

**Figure 8:**
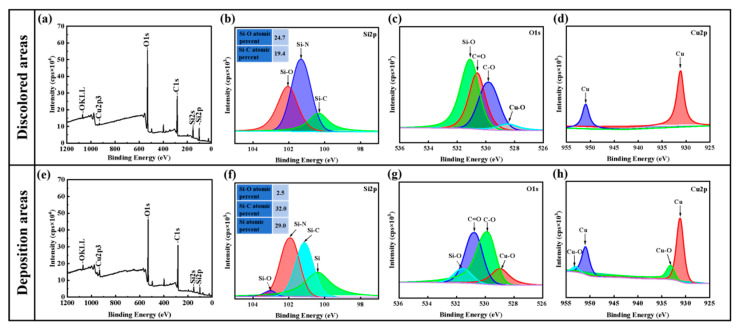
XPS analysis of modified areas on SiC surface: (**a**–**d**) discolored areas; (**e**–**h**) deposition areas.

The chemical state of modified regions on the SiC surface was further characterized by XPS, and the corresponding XPS spectra are presented in Figure 8. Figure 8a–c presents the XPS spectra of the discolored area. For the curve-fitted Si 2p spectrum shown in Figure 8a, three main peaks assigned to Si-O, Si-N, and Si-C bonds were identified, with the atomic concentrations of Si-O and Si-C bonds being 24.7% and 19.4%, respectively. Figure 8b displays the O 1s spectrum of the discolored area, which was dominated by Si-O bonds, with additional peaks corresponding to C=O, C-O, and Cu-O bonds. Figure 8c confirms the presence of elemental Cu in the discolored area via its Cu 2p spectrum. In contrast, Figure 8d–f presents the XPS spectra of the deposition area. As illustrated in the curve-fitted Si 2p spectrum in Figure 8d, the deposition area not only contained the three main peaks assigned to Si-O, Si-N, and Si-C bonds but also a prominent peak corresponding to elemental Si. The atomic percentages of these species differed significantly from those in the discolored area. The atomic percentage of Si-O bonds decreased to only 2.5%, while Si-C bonds and elemental Si were dominant at 32% and 29%, respectively. Correspondingly, Figure 8e shows that the content of Si-O bonds in its O 1s spectrum is much lower than that of C=O and C-O bonds. Furthermore, Figure 8f indicates the coexistence of elemental Cu and Cu-O bonds in the deposition area from its Cu 2p spectrum.

### 3.3. Measurement and Characterization of Interface Temperature During EDM Process

**Figure 9:**
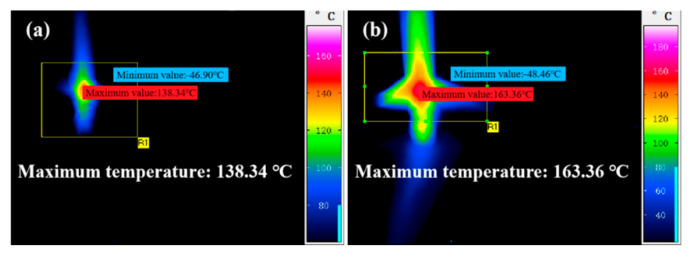
Observation results of high-speed infrared thermography at 10 V (**a**) and 15 V (**b**).

As shown in Figure 9, a high-speed infrared thermal imager was used to measure the interfacial temperature generated at the electrode tip during EDM in water under different voltages. The maximum temperatures recorded at 10 V and 15 V were 138.34 °C and 163.36 °C, respectively. However, the temperature of the plasma channel can reach 10,000–30,000 K during the discharge process [41]. Direct in situ measurement of such an ultra-high transient temperature is extremely challenging. In a water-mediated EDM environment, infrared temperature measurement suffers from inherent limitations, including insufficient spatial and temporal resolution, uncertain surface emissivity calibration, absorption and scattering effects caused by dielectric water and vapor bubbles, as well as the shielding effect of plasma. Meanwhile, the micro discharge channel possesses an extremely short lifetime, making it difficult for the infrared camera to capture the instantaneous peak temperature of the localized micro-region. Consequently, the measured temperature only reflects the macroscopic average temperature of the workpiece surface rather than the microscopic transient temperature of the discharge plasma zone.

**Figure 10:**
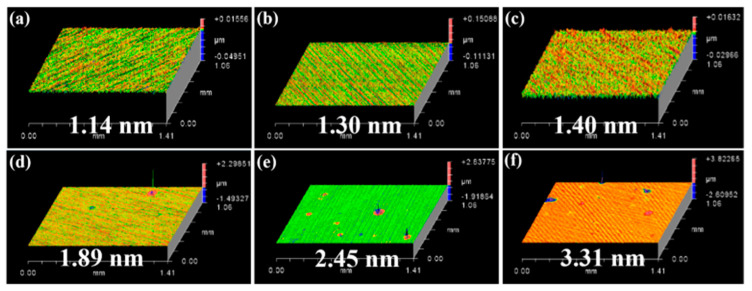
Surface morphologies and surface roughness of SiC substrates after ablation under different temperatures: (**a**) before ablation; (**b**) 250 °C; (**c**) 500 °C; (**d**) 750 °C; (**e**) 1000 °C; (**f**) 1250 °C.

Thus, a high-temperature ablation experiment was performed to further determine the onset oxidation temperature on the SiC surface. Figure 10 presents the surface morphologies and roughness of ablated SiC substrates at room temperature, 250 °C, 500 °C, 750 °C, 1000 °C and 1250 °C, with the corresponding values of 1.41, 1.30, 1.40, 1.89, 2.45 and 3.31 nm. The surface roughness of SiC substrates rose with increasing ablation temperature, owing to the formation of a surface oxidation layer.

**Figure 11:**
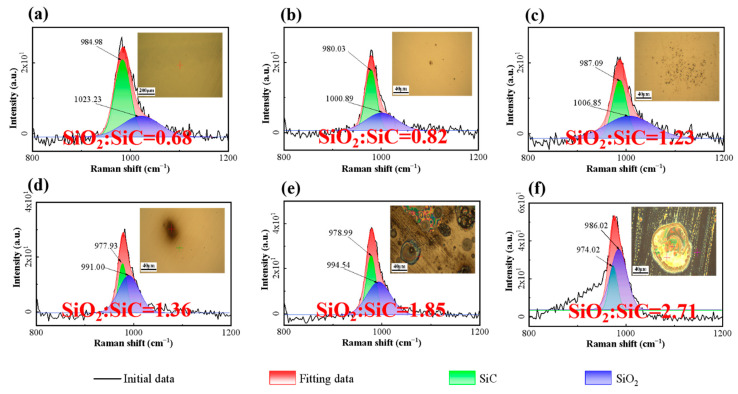
Surface morphologies and Raman detection results of SiC substrates before and after ablation at different temperatures: (**a**) before ablation; (**b**) 250 °C; (**c**) 500 °C; (**d**) 750 °C; (**e**) 1000 °C; (**f**) 1250 °C.

The surface morphologies and Raman detection results of SiC substrates after ablation at different temperatures are presented in Figure 11. Color spots started to form on the SiC surface at 250 °C, became dense at 1000 °C, and were completely covered by a colored oxide layer at 1250 °C. It can be confirmed that the temperature on the electrode tip in EDM exceeded 1000 °C based on the morphologies of SiC substrates processed by EDM. Raman spectroscopy indicated that the peak intensity of SiO_2_ increased significantly with the rise of ablation temperature.

### 3.4. COMSOL Simulation of the Interfacial Temperature Field

**Figure 12:**
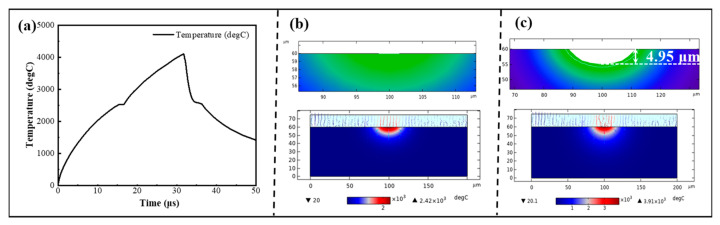
Temperature simulation results of the model under different processing times: (**a**) temperature variation curve of the model with processing times; crater morphologies and maximum temperatures of the model at 15 μs (**b**) and 32 μs (**c**).

A COMSOL temperature field simulation was conducted to study the interfacial state and the maximum surface temperature of SiC substrates under the application of a Gaussian heat source. The maximum interfacial temperature at different time steps is illustrated in Figure 12a, and the SiC substrate reached a maximum temperature of 3910 °C at 32 μs and then decreased with the increase of time steps. Figure 12b,c presents the SiC surface morphologies at 15 μs and 32 μs, respectively. Surface thermal craters initiated at 15 μs when the temperature reached 2410 °C. The interfacial temperature reached its peak at 32 μs, with the maximum material removal depth achieved on the SiC surface.

**Figure 13:**
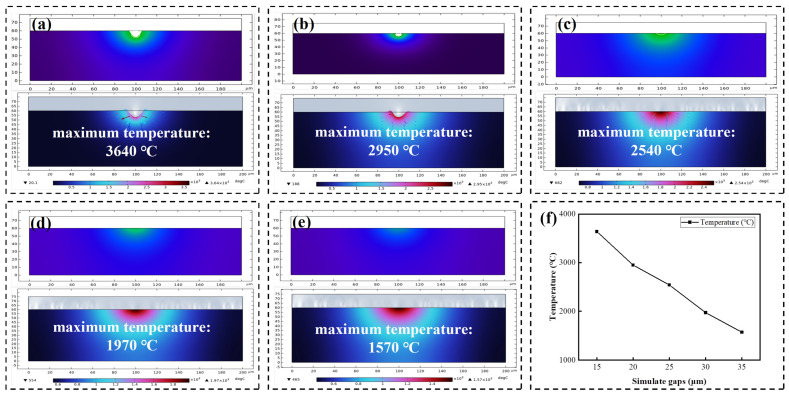
Temperature simulation results of the model under different processing gaps: crater morphologies and maximum temperatures at 15 μm (**a**), 20 μm (**b**), 25 μm (**c**), 30 μm (**d**) and 35 μm (**e**); (**f**) temperature variation curve of the model with processing gaps.

Figure 13 shows the temperature simulation results for different discharge gaps. Five gap parameters (15, 20, 25, 30, and 35 µm) were set to simulate the corresponding peak interface temperatures and crater morphologies, as depicted in Figure 13a–e. The result revealed that the peak interface temperature increased as the discharge gap decreased, and the resulting ablation craters became larger and deeper. This trend was attributed to higher heat concentration within smaller gaps, which impeded thermal dissipation and led to a more rapid rise in local temperature, ultimately causing higher peak temperatures and deeper craters.

**Figure 14:**
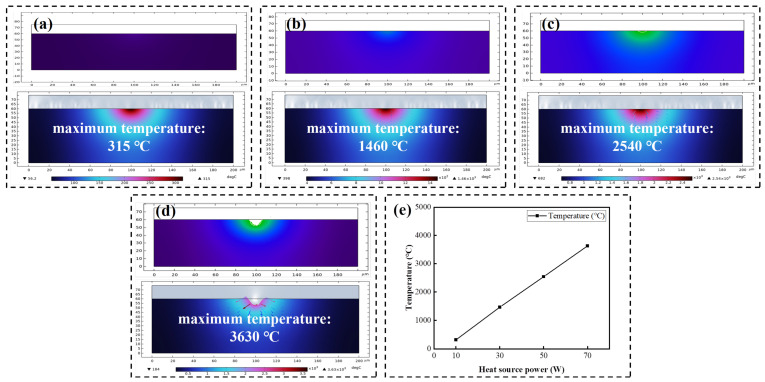
Temperature simulation results of the model at different heat source powers: crater morphologies and maximum temperatures at 10 W (**a**), 30 W (**b**), 50 W (**c**), and 70 W (**d**); (**e**) temperature variation curve of the model with heat source powers.

Figure 14 presents the temperature simulation results for various discharge power levels (10, 30, 50, 70 W). As shown in Figure 14a–d, discharge craters begin to form on the SiC surface when the interfacial temperature reaches 2540 °C at a power of 50 W. Figure 14e shows that the interface peak temperature rose with increasing discharge power. This demonstrates that higher discharge power caused a more intense thermal effect on the SiC surface, forming deeper crater morphologies.

**Figure 15:**
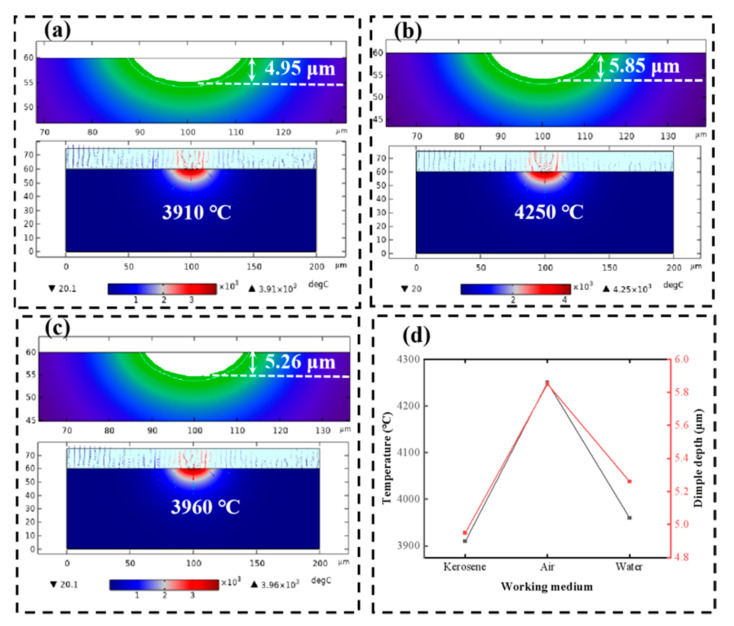
Temperature simulation results of the model at different working medium: crater morphologies and maximum temperatures at kerosene (**a**), air (**b**), and water (**c**); (**d**) variation curve of dimple depth and maximum temperature with working medium.

Figure 15 presents the temperature simulation results under different discharge medium conditions. The thermal effect on the SiC surface is the most pronounced in air, with both the peak temperature and crater depth being significantly higher than those in kerosene and water.

## 4. Discussion

The effects of EDM process parameters on the modification behavior of SiC substrates were systematically investigated, with a focus on the impacts of processing time, discharge voltage, inter-electrode gap, and machining medium on the surface morphologies of SiC substrates. The crater depth did not increase continuously but fluctuated within a certain range as processing time increased, which indicates that the vertical material removal capacity reached saturation at the initial discharge stage due to the accumulation of erosion residues, which hinders the effective transfer of discharge energy to the workpiece. In contrast, the height of edge protrusions increased gradually with increasing processing time and then tended to stabilize, revealing that the accumulated molten material could not be effectively expelled but instead accumulated and solidified at the crater edges. Overall, prolonging the processing time expanded the machining range and increased edge accumulation to a certain degree, yet it contributed little to the increase in crater depth. The crater depth increased steadily with the increase in discharge voltage, demonstrating that a higher single-pulse energy induced a deeper material removal. Meanwhile, the height of the protrusions increased sharply, with the most significant growth observed under moderate voltage conditions, followed by a slowdown in the growth rate. This suggests that the discharge energy not only determines the amount of material removed but also dominates the scale of molten material ejection and resolidification at the crater edges. The interelectrode gap had an insignificant effect on the modification depth. The crater depth remained constant at approximately 100 μm with the increase in interelectrode gap. However, the height of the edge protrusions exhibited a distinct U-shaped trend (decreasing first and then increasing) with the variation in interelectrode gap, since an excessively large or small gap may lead to energy dispersion of the discharge channel or difficulty in debris removal, thus exacerbating the accumulation of the recast layer [42]. The state of machining dielectric exerts a decisive effect on the erosion and accumulation behaviors. The maximum crater depth and edge protrusion height were obtained in the air medium. The extremely high protrusion-to-depth ratio reveals that the gaseous dielectric failed to provide a sufficient explosive impact force to eject the molten metal from the substrate, resulting in the displacement of the vast majority of the molten material and its subsequent cooling and accumulation at the edges. In contrast, the impact force generated by vaporization in liquid dielectrics can effectively expel the molten material and significantly inhibit the accumulation of the recast layer [43]. Among the liquid dielectrics, kerosene yielded the lowest protrusion height, which exhibited a superior debris-removal capacity to water. In conclusion, liquid dielectrics can effectively suppress edge protrusions and achieve an optimal surface finish.

The surface of SiC substrates after EDM is clearly divided into two visually distinct regions: a dark-hued loosely agglomerated deposition zone at the discharge center and a surrounding discoloration zone with variable luster and coloration. The remarkable differences in morphology and color between these two regions correspond to distinct modes of material removal or transformation, respectively. Results from Raman spectroscopy and XPS analyses demonstrate that SiC substrates are primarily oxidized to SiO_2_ in the discoloration zone with minimal thermal influence, whereas the black deposition zone is subjected to more concentrated thermal energy, with SiC decomposing to form elemental silicon and carbon mixed with copper oxide deposits originating from the electrode. Meanwhile, deionized water acts not only as a cooling and dielectric medium but also undergoes dissociation under high-temperature plasma discharge to produce active species such as H·, OH· and oxygen-containing radicals. These reactive radicals participate in the plasma-assisted oxidation of SiC, further affecting the oxidation reaction kinetics and the generation of SiOx/SiO_2_-rich surface layers [44]. Compared with the dense and chemically inert pristine SiC substrate, the modified layer features lower hardness, higher chemical activity and loose local microstructure. These characteristics can reduce the mechanical removal resistance, which fundamentally facilitates material removal and surface planarization. The Cu 2p XPS spectra of the two regions reveal that the electrodeposited copper species exist in two chemical forms, namely metallic copper (Cu) and copper oxide (Cu-O), respectively. This phenomenon indicates that the copper electrode erosion occurs during this process, and the eroded material redeposits onto the SiC workpiece surface. The formed copper-containing surface layer can effectively modify the local surface conductivity of the workpiece and redistribute the near-surface electric field. In addition, a Schottky junction tends to form at the interface between metallic copper and semiconductor silicon carbide, which further changes the local charge distribution and interfacial charge transport characteristics. These variations further affect the breakdown position, migration law and spatial randomness of subsequent discharge points, exerting potential influences on the material removal characteristics and surface microstructure evolution of SiC.

For temperature characterization, the maximum transient temperature measured directly via a high-speed infrared thermal imager was only 163.36 °C. To more accurately quantify the temperature threshold for thermal oxidation of SiC, a series of high-temperature ablation experiments was conducted over a temperature range of 250 °C to 1250 °C. With the increase in temperature, the surface morphologies of SiC substrates evolved from a smooth state to the appearance of black dots and spots, and eventually became fully covered by a discoloration zone. An increase in temperature was positively correlated with the rise in SiC surface roughness and the degree of oxidation. Notably, the furnace heating ablation experiment cannot be simply treated as a direct equivalent simulation of the EDM process. Furnace oxidation proceeds under quasi-static and near-equilibrium thermal conditions, while EDM discharge is a microsecond-scale, highly non-equilibrium process characterized by localized plasma action, impact shock wave, ultrafast temperature rise, and rapid quenching. Considerable differences exist between the two processes in terms of thermal diffusion length, oxidation kinetics, ambient medium environment, and surface reaction pathways. Accordingly, the furnace experimental results are only suitable to provide a qualitative reference for the thermal oxidation threshold of SiC. COMSOL simulations with different step sizes indicated that crater initiation occurred on the SiC model surface when the temperature reached a specific temperature threshold. The effects of discharge power, electrode gap and machining medium on the interfacial temperature and surface morphologies of SiC substrates were investigated via COMSOL simulations. A reduction in discharge gap or an increase in discharge power both led to a rise in the interface peak temperature. Surface thermal crater initiation occurred on the SiC surface when the temperature reached the critical value. The dimensions of craters were positively correlated with the interface temperature. Both the temperature and crater dimensions in the air medium were significantly larger than those in water and kerosene media.

**Figure 16:**
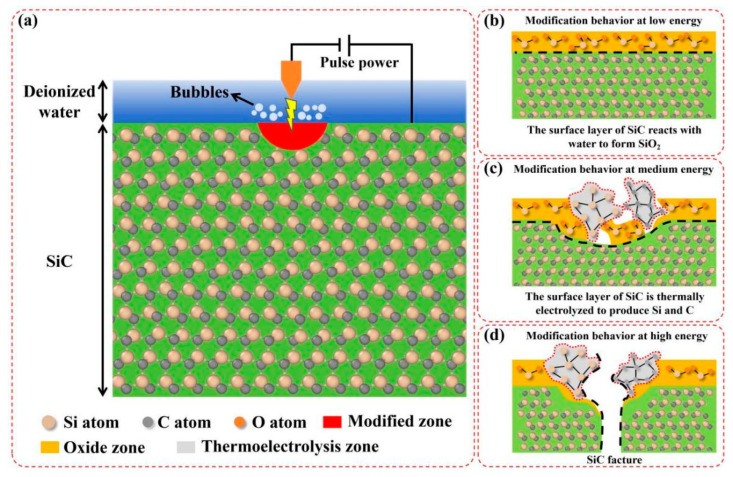
Schematic diagram of the chemical modification mechanism of SiC substrates during EDM (**a**); Chemical modification behaviors of the modified zones on the SiC surface under low (**b**), moderate (**c**), and high (**d**) discharge energy.

Figure 16 illustrates the chemical modification mechanism induced on the SiC surface during EDM. As shown in Figure 16a, the electrical discharge between the electrode and workpiece driven by a pulsed power supply generates an instantaneous high temperature in the local area of the SiC substrate, thereby forming a modified zone on the SiC surface. Figure 16b–d further reveals the chemical modification mechanisms on the SiC surface under different discharge energy levels. The chemical reaction of the modified zone on the SiC surface under low discharge energy is depicted in Figure 16b. The surface of the SiC substrate reacts with water to form SiO_2_, thus generating a soft oxide layer on the SiC surface. As shown in Figure 16c, the SiC substrate decomposes into crystalline silicon and graphitic carbon with the increase in discharge energy under moderate discharge energy conditions. As presented in Figure 16d, the SiC substrate undergoes breakdown and fracture under high discharge energy conditions. The clarification of this mechanism lays a solid theoretical foundation for the process optimization of EDM for SiC substrates and provides key guidance for damage control in the EDM of SiC.

## 5. Conclusions

In this study, the chemical modification mechanism on the SiC surface in the EDM process was systematically investigated. The influences of distinct discharge parameters on the surface morphologies of SiC substrates were analyzed in detail, while the surface states and chemical compositions of SiC substrates under different discharge energy levels were characterized. Furthermore, the spark discharge temperature required to induce prominent thermal oxidation of SiC substrates was quantified through ablation experiments. The experimental results were further validated via COMSOL temperature field simulations under various parameter conditions. The key conclusions drawn from this work are summarized as follows:

(1) Discharge voltage is the dominant factor determining the surface morphologies of SiC substrates. The surface state of SiC substrates evolves from the formation of a discoloration zone to an agglomeration deposition zone, and surface cracking eventually occurs with the increase in discharge voltage. The depth of SiC surface modification gradually tends to saturation as the processing time is prolonged. A proper discharge gap facilitates favorable chemical modification on the SiC surface, while an excessively small or large gap will lead to the formation of deposits. In comparison with air as a discharge medium, the liquid dielectric yields a better modification surface.
(2) Varied discharge energies induce distinct chemical modifications on the SiC surface. Specifically, low-energy discharge primarily oxidizes the SiC surface to SiO_2_, whereas high-energy discharge electrolyzes SiC into crystalline silicon and graphitic carbon.
(3) The critical temperature threshold for the prominent thermal oxidation on the SiC surface was determined to be 1000 °C. During the thermal oxidation stage, the oxidation degree of the SiC surface increases with the rise in the interfacial temperature.
(4) The interfacial temperature of SiC substrates increased with increasing power input. A narrower machining gap was associated with a higher interfacial temperature of SiC substrates. Furthermore, SiC processed in an air dielectric exhibited a higher interfacial temperature than that machined in water or kerosene dielectrics. The discharge crater began to form on the SiC surface when a certain temperature threshold was reached, and its depth increased as the temperature rose.

## Figures and Tables

**Table 1:** Experimental parameters.

Parameter Name	Value
Pulse frequency	20 k Hz
Pulse duty cycle	90%
Machining Time	10, 20, 30, 40, 50, 60 s
Machining Voltage	1, 3, 5, 10, 15, 20 V
Machining gap	10, 30, 50, 70, 90 µm
Machining medium	air, deionized water, kerosene

**Table 2:** Simulation conditions.

Parameter Name	Value
Discharge power	10–70 W
Discharge duration	30 µs
Total simulation time	50 µs
Discharge gap	15–55 µm
Initial radius of discharge channel	0.1 µm
Final radius of discharge channel	100 µm
Linear expansion time of discharge channel	2 µs
Energy distribution coefficient (Anode)	41.2% [38]

**Table 3:** Thermophysical properties of media and anode material.

Parameter Name	Value			
	Air	Water	Kerosene	SiC
Density (kg/m^3^)	1.184	997	810	3210
Thermal conductivity (W/m/K)	0.026	0.6	0.15	370
Solid specific heat capacity (J/kg/K)	1005	4180	2150	710
Coefficient of thermal expansion (K^−1^)	3.3 × 10^−3^	2.1 × 10^−4^	9 × 10^−4^	4.5 × 10^−6^
Latent heat of fusion (J/kg)	-	3.3 × 10^5^	-	1.06 × 10^8^
Melting point tm (K)	43	273	233	3003
Evaporation point tv (K)	-	373	473	3173
Darcy’s law coefficient c/d	-	-	-	1 × 10^6^/1 × 10^−3^

## Data Availability

The original contributions presented in this study are included in the article. Further inquiries can be directed to the corresponding authors.

## References

[1] Li Y., Zhang Z., Song Q., Shi H., Hou Y., Yue S., Wang R., Cai S., Zhang Z. (2024). Surface micromorphology and nanostructures evolution in hybrid laser processes of slicing and polishing single crystal 4H-SiC. J. Mater. Sci. Technol..

[2] Tian Z., Chen X., Xu X. (2020). Molecular dynamics simulation of the material removal in the scratching of 4H-SiC and 6H-SiC substrates. Int. J. Extrem Manuf..

[3] Chen G., Li J., Long J., Luo H., Zhou Y., Xie X., Pan G. (2021). Surface modulation to enhance chemical mechanical polishing performance of sliced silicon carbide Si-face. Appl. Surf. Sci..

[4] Gao G., Wang H., Li R., Zhang B., Ma W., Xiang D., Ma J. (2025). Modeling of axial force and torque in UAD of polycrystalline 3C-SiC. Int. J. Mech. Sci..

[5] Tuci G., Liu Y., Rossin A., Guo X., Pham C., Giambastiani G., Pham-Huu C. (2021). Porous silicon carbide (SiC), a chance for improving catalysts or just another active-phase carrier. Chem. Rev..

[6] Xu C., Song X., Li N., Su Y., Kang R., Gao S. (2025). First-principles insights into the synergistic chemical-mechanical removal mechanism of 4H-SiC in chemical mechanical polishing. Appl. Surf. Sci..

[7] Han X., Jin Z., Mu Q., Yan Y., Zhou P. (2022). Morphological characteristics and formation mechanism of latent scratches in chemical mechanical polishing. J. Mater. Process. Technol..

[8] Wang L., Wu R., Niu L., An Z., Jin Z. (2022). Study on electrochemical mechanical polishing process of silicon carbide crystal. Diam. Abras. Eng..

[9] Chen S., Li W., Li X., Yang W. (2019). One-dimensional SiC nanostructures, Designed growth, properties, and applications. Prog. Mater. Sci..

[10] Beaucamp A., Simon P., Charlton P., King C., Matsubara A., Wegener K. (2017). Brittle-ductile transition in shape adaptive grinding (SAG) of SiC aspheric optics. Int. J. Mach. Tools Manuf..

[11] Alam M.N., Siddiquee A.N., Khan Z.A., Khan N.Z. (2022). A comprehensive review on wire EDM performance evaluation. Proc. Inst. Mech. Eng. Part E J. Process Mech. Eng..

[12] Li Z., Tang J., Bai J. (2020). A novel micro-EDM method to improve microhole machining performances using ultrasonic circular vibration (UCV) electrode. Int. J. Mech. Sci..

[13] Dong H., Li R., Zhang Q., Zhou J. (2025). High-accuracy and high-surface-quality electrical discharge machining using bipolar hybrid pulses. J. Mater. Process. Technol..

[14] Gong S., Zhang L., Hu Y., Chan K., Liu J., Wang Z., Wang Y. (2025). Micro-holes machining characteristics of Cf-ZrB_2_-SiC by Micro-EDM and taper control based on swing device. Ceram. Int..

[15] Wu X., Liu Y., Qi L., Li D., Ma C., Ji R. (2024). Electrical discharge and arc milling with automatic tracking of optimal flushing direction, A novel high-efficiency compound machining method. Chin. J. Aeronaut..

[16] Saxena K.K., Agarwalb S., Khare S.K. (2016). Surface characterization, material removal mechanism and material migration study of micro EDM process on conductive SiC. Procedia CIRP.

[17] Selvarajan L., Venkataramanan K. (2023). Surface morphology and drilled hole accuracy of conductive ceramic composites Si3N4-TiN and MoSi_2_-SiC on EDMed surfaces. Wear.

[18] Selvarajan L., Venkataramanan K., Nair A., Srinivasan V. (2023). Simultaneous multi-response Jaya optimization and Pareto front visualization in EDM drilling of MoSi_2_-SiC composites. Expert Syst. Appl..

[19] Cao H.T., Ho J.R., Tung P.C., Tsui H.P., Lin C.K. (2025). Characterization of machined surface in semi-conductive SiC wafer subjected to micro-EDM drilling. Mater. Sci. Semicond. Process..

[20] Singh M.A., Joshi K., Hanzel O., Singh R.K., Šajgalík P., Marla D. (2020). Identification of wire electrical discharge machinability of SiC sintered using rapid hot pressing technique. Ceram. Int..

[21] Zhao Y., Kunieda M., Abe K. (2018). A novel technique for slicing SiC ingots by EDM utilizing a running ultra-thin foil tool electrode. Precis. Eng..

[22] Liu Y.H., Ji R., Li Q., Yu L., Li X. (2009). An experimental investigation for electric discharge milling of SiC ceramics with high electrical resistivity. J. Alloys Compd..

[23] Mohan B., Rajadurai A., Satyanarayana K. (2002). Effect of SiC and rotation of electrode on electric discharge machining of Al-SiC composite. J. Mater. Process. Technol..

[24] Jin L., Gong Y., Zhu R., Hu Y., Liu M. (2024). Experimental study of micro-prismatic electrode array wear during EDM and application to the preparation of microcylindrical electrode array. Wear.

[25] Jiang B., Lan S., Ni J., Zhang Z. (2014). Experimental investigation of spark generation in electrochemical discharge machining of non-conducting materials. J. Mater. Process. Technol..

[26] Lei J., Shen H., Wu H., Pan W., Wu X., Zhao C. (2024). Ultrasonic vibration-assisted electrical discharge machining of enclosed microgrooves with laminated electrodes. J. Mater. Res. Technol..

[27] Jiang K., Wu X., Lei J., Hu Z., Gao G., Tang Y., Diao D. (2021). Investigation on the geometric evolution of microstructures in EDM with a composite laminated electrode. J. Clean. Prod..

[28] Yoo H.K., Ko J.H., Lim K.Y., Kwon W.T., Kim Y.W. (2015). Micro-electrical discharge machining characteristics of newly developed conductive SiC ceramic. Ceram. Int..

[29] Shabgard M., Badamchizadeh M., Ranjbary G., Amini K. (2013). Fuzzy approach to select machining parameters in electrical discharge machining (EDM) and ultrasonic-assisted EDM processes. J. Manuf. Syst..

[30] Wang C., Sasaki T., Hirao A. (2024). Bubble behavior of single-pulse discharge in EDM. J. Manuf. Process..

[31] Zhang Y., Liu Y., Shen Y., Ji R., Li Z., Zheng C. (2014). Investigation on the influence of the dielectrics on the material removal characteristics of EDM. J. Mater. Process. Technol..

[32] Bian R., Liu Y., Sun A., Wu X., Liu P., Zheng C., Han Y., Zhang P., Zhang M. (2025). High-performance and sustainable electrical discharge machining with green microdroplet dielectric. J. Mater. Res. Technol..

[33] Gong S., He X., Wang Y., Wang Z. (2022). Material removal mechanisms, processing characteristics and surface analysis of Cf-ZrB_2_-SiC in micro-EDM. Ceram. Int..

[34] Guan J., Zhao Y. (2022). Non-contact grinding/thinning of silicon carbide wafer by pure EDM using a rotary cup wheel electrode. Precis. Eng..

[35] Guan J., Zhao Y. (2022). Dual-wafer intergrinding thinning by bipolar-discharge EDM with a capacity-coupled pulse generator considering large gap capacitance and minimization of discharge energy. Results Eng..

[36] Yan J., Tan T. (2015). Sintered diamond as a hybrid EDM and grinding tool for the micromachining of single-crystal SiC. CIRP Ann.—Manuf. Technol..

[37] Qu J., Zhang S., Zhang W., Wang Z., Sha Z., Liu Y. (2026). Mechanisms of erosion and material removal in EE-EDM of PRMMCs. Compos. Part A Appl. Sci. Manuf..

[38] Xia H., Kunieda M., Nishiwaki N. (1996). Removal amount difference between anode and cathode in EDM process. Int. J. Electr. Mach..

[39] Wang S., Li X., Yue S., Zhang M., Zhu B., Zhao P., Zhou M., Zhao H. (2025). Continuous phase transformation in monocrystalline silicon during indentation. Appl. Surf. Sci..

[40] Wang B., Melkote S.N., Saraogi S., Wang P. (2020). Effect of scratching speed on phase transformations in high-speed scratching of monocrystalline silicon. Mater. Sci. Eng. A.

[41] Liu C., Li Q., Yang X. (2023). Analysis of arc plasma characteristics and energy distribution in EDM based on two-temperature model. Precis. Eng..

[42] Saxena K., Srivastava A., Agarwal S. (2016). Experimental investigation into the micro-EDM characteristics of conductive SiC. Ceram. Int..

[43] Peng W., Tang J., Li Z. (2025). Influence of dielectric characteristics on bubble dynamics and material removal mechanism in electrical discharge machining. Phys. Fluids.

[44] Chen S., Lei H. (2026). Microscopic chemical mechanism of hydroxyl radical-enhanced SiC planarization efficiency revealed by ReaxFF molecular dynamics simulation. Appl. Surf. Sci..

